# Association Between Unaided Speech Perception in Noise and Hearing Aid Use Mediated by Perceived Benefit

**DOI:** 10.3390/audiolres15030050

**Published:** 2025-05-01

**Authors:** Anthony Marcotti, Catherine Silva-Letelier, Javier Galaz-Mella, Alejandro Ianiszewski, Nicole B. Vargas, Eduardo Fuentes-López

**Affiliations:** 1 Escuela de Fonoaudiología, Facultad de Ciencias de la Rehabilitación y Calidad de Vida, Universidad San Sebastián, Santiago 7510157, Chile; anthony.marcotti@uss.cl (A.M.); alejandro.ianiszewsk@uss.cl (A.I.); 2 Magister en Epidemiología, Escuela de Salud Pública, Facultad de Medicina, Pontificia Universidad Católica de Chile, Santiago 8330023, Chile; cpsilva5@uc.cl; 3 Faculty of Rehabilitation Sciences, School of Speech Therapy, Exercise and Rehabilitation Sciences Institute, Universidad Andres Bello, Santiago 7591538, Chile; jigalaz@alumni.uc.cl; 4 Facultad de Salud y Ciencias Sociales, Universidad de Las Américas, Viña del Mar 2531098, Chile; nvargas9@edu.udla.cl; 5 Departamento de Fonoaudiología, Escuela de Ciencias de la Salud, Facultad de Medicina, Pontificia Universidad Católica de Chile, Santiago 7820436, Chile

**Keywords:** hearing aids, speech perception in noise, perceived benefit, older adults, auditory rehabilitation, mediation analysis

## Abstract

**Background/Objectives**: The conventional strategy for addressing age-related hearing loss is hearing aid (HA) use, yet many individuals underutilize their devices. Despite the positive effects of HA use, adherence remains low, highlighting the importance of studying associated variables. We hypothesize that better unaided speech perception in noise (SPiN) would be associated with greater perceived benefit from HAs, which, in turn, would be linked to increased HA use. **Methods**: A cross-sectional study design was used, including 114 older adults (≥65 years) who were HA users. HA use and perceived benefit were assessed using questions 1 and 2 of the International Outcome Inventory for Hearing Aids (IOI-HAs), while unaided SPiN performance was measured monaurally with a speech-in-noise test. In the mediation analysis, SPiN performance was the predictor, perceived benefit the mediator, and HA use the outcome. Direct and indirect effects were evaluated using generalized structural equation modeling. **Results**: No significant total effect was found for the right ear. For the left ear, there was a significant indirect effect of SPiN performance on HA use through perceived benefit (OR = 1.26, 95% CI 1.06–1.57, *p* = 0.019) but no direct effect (*p* = 0.563). In addition, a significant total effect of left ear SPiN performance on HA use was observed (*p* = 0.041). **Conclusions**: The findings suggest that unaided SPiN performance—particularly in the left ear—may be indirectly associated with HA use through its effect on perceived benefit. These results underscore the potential value of including SPiN assessments in the HA fitting process and counseling strategies for older adults.

## 1. Introduction

The World Health Organization (WHO) estimates that 25% of older adults worldwide have disabling hearing loss [1]. Age-related hearing loss (ARHL) is a prevalent and potentially modifiable risk factor for adverse health outcomes. It is among the leading contributors to disability-adjusted life years (DALYs) [2] and is associated with increased social isolation [3], higher odds of depression [4], increased risk of falls [5], cognitive impairment [6], and dementia [6,7].

The conventional strategy for addressing ARHL is the use of hearing aids (HAs) [8,9,10]. Consistent utilization of HAs over time has been significantly associated with improvements in communication [11], generic and specific quality of life [12], and delayed onset of cognitive decline among individuals at risk [13]. Despite these benefits, the proportion of individuals prescribed HA who choose not to use them varies widely, ranging from 1% to 57% [14]. Furthermore, among those who do use them, up to 15% wear them for less than an hour a day [15]. Therefore, investigating the variables associated with HA non-use remains highly relevant.

The hours per day that a person uses their HA is commonly referred to as HA use [16]. Longer usage time is associated with a reduction in participation restrictions, better quality of life, and increased satisfaction and benefits with HA [17]. However, not all older adults who use HAs perceive benefits from their use [18]. Perceived benefit with HAs refers to the subjective assessment of a change in hearing function or communication ability obtained with the HAs [19]. Perceived benefit is associated with HA use [20,21], and, specifically among older adults, the lack of perceived benefit has been identified as one of the main reasons for HA abandonment [22,23,24].

Both HA use and perceived benefit with HAs are determined by a complex interplay of factors, as highlighted in a recent systematic review by Mothemela et al. [25]. This review identified several audiological factors, such as degree of hearing loss and type of HA fitting, and non-audiological factors, including age, cognitive status, social support, and self-efficacy with HA. Some of the variables identified, such as the degree of hearing loss, show associations with both HA use and perceived benefit with HAs, while others, for example, HA self-efficacy, have been linked only to HA use. Overall, these findings underscore the need to account for a broad range of factors when assessing and optimizing HA outcomes.

Among the various factors influencing HA use and perceived benefit, speech recognition ability is particularly noteworthy [25]. In older adults, difficulties in speech perception tend to increase with age, irrespective of ARHL severity [26]. Several studies have linked better unaided speech perception in quiet conditions to better perceived benefit and increased HA use [20,21,27,28]. Houmøller et al. [20], Wang et al. [21], and Chang et al. [27] demonstrated that higher word recognition scores (WRS)—that is, better speech perception—were associated with higher total scores on the International Outcome Inventory for Hearing Aids (IOI-HAs), which includes questions to assess both use and perceived benefit. Higher IOI-HAs scores indicate better outcomes in the domains evaluated by the instrument. In Wang et al.’s [18] study, the same association was found between WRS and Factor 1 of the IOI-HAs, which includes questions on use, perceived benefit, satisfaction, and quality of life. Additionally, Wu et al. [28] found that the better results of a speech recognition test were associated with both the Factor 1 score and the total score of the IOI-HAs.

However, despite the associations observed with speech perception tests conducted in quiet conditions, some studies have found that these relationships with HA use and perceived benefit outcomes are not sustained over time. Chang et al. [27] found this association in new HA users only one-month post-fitting but not after three months. A possible explanation for this is that speech perception measured in quiet conditions has low ecological validity and does not account for the complexities of real-world listening situations, such as background noise [29].

Older adults experience a decline in hearing performance as they age, which is particularly noticeable in their ability to understand speech in noisy environments [30]. Although the severity of ARHL is a key predictor of performance on tasks involving speech perception in noise (SPiN), research shows that age independently influences these outcomes [31]. In fact, even when controlling for the degree of ARHL, older adults perform significantly worse on behavioral SPiN tasks and report greater difficulties in noisy settings compared to younger adults [32]. These difficulties persist even with the use of HAs [33]. Given that challenges in SPiN can substantially impact daily communication, SPiN tests may offer a more accurate reflection of the real-world benefits that users could perceive with their HAs.

Walden and Walden [34] found that better performance on the unaided QuickSIN test in free-field conditions was associated with higher scores on the Hearing Aid Usefulness Scale (HAUS), an instrument that assesses patients’ overall perception of the usefulness of their HAs in daily life, where higher scores indicate greater perceived usefulness. The QuickSIN test is a speech-in-noise measure that presents sentences embedded in fixed levels of multi-talker babble, allowing for the estimation of signal-to-noise ratio (SNR) loss based on the number of key words correctly repeated, where a lower SNR loss reflects better speech-in-noise perception [35].

On the other hand, in Mendel’s [36] study, better performance on unaided SPiN measures—specifically, the Revised Speech Perception in Noise (R-SPIN), the Hearing in Noise Test (HINT), and the QuickSIN—was correlated with higher scores in certain dimensions of the Hearing Aid Performance Inventory (HAPI), an instrument designed to evaluate perceived benefit with HAs in various listening situations. Higher scores on the HAPI indicate greater perceived benefit. The R-SPIN test evaluates speech perception in noise by presenting sentences with high- and low-predictability contexts, where high-predictability sentences provide contextual cues to aid in understanding, while low-predictability sentences rely more on auditory processing [37]. In contrast, the HINT is an adaptive test that determines the signal-to-noise ratio (SNR) at which 50% of sentences are correctly repeated, using spectrally matched noise presented at a fixed level from different azimuth angles while adjusting speech intensity [38]. Together, these tests provide a comprehensive evaluation of speech perception in noise, which appears to be closely linked to the perceived benefits of HA.

The findings of Walden and Walden’s [34] and Mendel’s [36] studies suggest that unaided SPiN performance may predict the perceived benefits of HA. Walden and Walden [34] studied HA users with 2 months to 20 years of experience (mean = 5.3 years). Mendel [36], on the other hand, examined users with 6 months to 6 years of experience (mean = 1.7 years), suggesting that this association holds for both new and experienced users. However, the relationship between SPiN performance and actual HA use was not investigated.

Based on the findings described above, better unaided SPiN performance is associated with greater perceived benefit from HAs [34,36]. In turn, greater perceived benefit is associated with increased HA use [20,21]. However, previous research has not explicitly examined the potential mediated relationships among these variables. It is possible to hypothesize that better-unaided SPiN performance could be indirectly associated with increased HA use through greater perceived benefit. Therefore, the primary objective of the present study was to evaluate the direct and indirect associations—through perceived benefit with HAs—between unaided SPiN performance and HA use. Additionally, as observed with speech perception in quiet conditions [21,28], it is possible to hypothesize that there is, in turn, a direct association between SPiN performance and HA use. Consequently, the secondary objective was to examine the direct association between unaided SPiN performance and HA use. This information could aid clinicians in the HA fitting process, especially when counseling new HA users regarding realistic expectations.

## 2. Materials and Methods

### 2.1. Study Design

The present study is a secondary data analysis of the clinical trial ISRCTN54021189 available at https://www.isrctn.com/ISRCTN54021189 (accessed on 3 March 2025). A cross-sectional analysis of the baseline measurements from the clinical trial was conducted. The study protocol received approval from the Ethical Scientific Committees at the Facultad de Medicina of the Pontificia Universidad Católica de Chile (180405001), Servicio de Salud Metropolitano Sur-Oriente (NO2886), and Servicio de Salud Metropolitano Sur (23-25042019) of the Metropolitan Region, Chile. All the participants provided informed consent. This study was reported in accordance with the Strengthening the Reporting of Observational Studies in Epidemiology (STROBE) guidelines [39].

Participants were recruited between October 2019 and October 2020, and evaluations were carried out between December 2019 and December 2020. All the assessments took place at Family Health Centers (CESFAM in Spanish), the most common type of primary health care (PHC) establishment in the Chilean public health system. Four centers from the municipalities of Puente Alto, San Bernardo, and San Joaquín in the Metropolitan Region, and one center from the municipality of Valparaíso in the Valparaíso Region participated in the study. The study anticipated no harm, adverse effects, or risks for the participants.

### 2.2. Participants

All the participants were older adults aged 65 or above who had received HAs through the Chilean public health system’s Explicit Health Guarantees (GES) within the five years preceding the study. To be included in the study, participants needed to possess at least one functional HA. Additionally, they were required to have a normal cognitive status, demonstrated by a score of ≥22 on the Chilean-validated version of the Mini-Mental State Examination (MMSE) [40], which has previously been used in studies with similar populations [41], and no communication disorders of neurological origin.

The GES public policy provides HAs either for free or with a maximum copayment of 20%, depending on the health coverage of the beneficiary, to all adults aged 65 or older with bilateral hearing loss and a pure tone average (PTA) of ≥40 dB HL at the frequencies of 0.5, 1.0, 2.0, and 4.0 kHz [38]. Exceptionally, this policy also includes individuals with a PTA of ≥35 dB HL in the better ear, who have at least mild/moderate hearing difficulty, as evidenced by a score of ≥10 on the Shortened Hearing Handicap Inventory for the Elderly (HHIE-S). Health services operating under this policy are required to procure HAs through public tenders targeted at private companies. The HAs obtained through these tenders are of low range, with an average cost of USD 250 per unit, and have a minimum of 8 channels and up to 4 programs. All the devices are digital behind-the-ear (BTE) models, with no variability in device type or core technology across users. Minor differences, such as silicone or acrylic earmolds, are determined by audiological criteria and do not represent functional heterogeneity. The standard GES program provision includes only one HA, regardless of whether hearing loss is bilateral. However, if after one year the patient demonstrates consistent device usage, the GES program contemplates the provision of a second HA. Despite this regulation, it is common for patients to be unaware that they may qualify for a second device, resulting in continued use of only one HA. Thus, most of the study samples received monaural fittings. In practice, when only one device is provided, it is often placed in the right ear for practical reasons, as most users are right-handed and find it easier to manipulate the device on that side.

The list of beneficiaries of the GES policy from the past five years was accessed at each participating PHC center. Out of a total of 516 potential participants, 261 were assessed for eligibility. The remaining 255 were not evaluated because they could not be contacted (n = 116), declined to participate (n = 57), had moved to another city (n = 44), their families reported their death (n = 17), or for other reasons (n = 21). Of the subjects assessed for eligibility, 147 were excluded due to cognitive impairment (n = 48), malfunctioning HA (n = 39), lost HA (n = 17), and other reasons (n = 12). Additionally, some subjects decided not to participate after being assessed for eligibility (n = 31). Ultimately, 114 subjects participated in this study.

### 2.3. Variables

#### 2.3.1. Predictor: Speech Perception in Noise (SPiN)

For the evaluation of SPiN, the Speech in Noise (SIN) test from the Santiago APD auditory processing battery [42] was used. This test consists of 4 lists of 25 monosyllabic words presented monaurally with ipsilateral white noise. Each ear was evaluated with 2 lists: one with a signal-to-noise ratio (SNR) of 10 dB and another at 0 dB. The test was administered at an intensity of 40 dB HL above the PTA, and the subject’s task was to repeat the words they heard. Each correctly repeated word was scored as 4%, with a maximum performance score of 100%. For each ear, a single SPiN score was calculated as the average of the performances from the 10 dB and 0 dB SNR lists. This test was recorded in mp3 format with a quality of 320 kbps and played on a from Samsung Galaxy Note 10.1 Tablet (Seoul, South Korea), connected to the CD input of the audiometer using a 2 RCA to 3.5 mm Mini Jack cable. A Resonance^®^ r27a audiometer (Gazzaniga, Bergamo, Italy) with Telephonics TDH-39 headphones (Santa Ana, CA, USA) was used. Calibration was performed according to the instructions of the Santiago APD battery by Auditec Inc (St. Louis, MO, USA) [42]: a track containing a 1000 Hz pure tone included in the test material was used to adjust the audiometer’s VU meter to 0 dB, using channel 1 for the left ear and channel 2 for the right ear. In the absence of a soundproof booth, a consultation room at the participating establishments was designated and exclusively equipped for this study. Evaluations were scheduled during times when the ambient noise level did not exceed 45 dBA, as measured by an Amprobe SM-10 (Everett, WA, USA) sound level meter [43]. All the participants were assessed under these same conditions. Additionally, all the participants had already initiated HA use at the time of the SPiN testing.

Since the evaluations were conducted in PHC facilities rather than in controlled audiological environments, free-field testing was not feasible due to the lack of sound-treated rooms, calibrated speaker systems, and spatial setups typically required for accurate presentation [44]. In this context, monaural testing via headphones allowed for greater control over the presentation and enabled ear-specific performance to be assessed—particularly relevant given the variability in participants’ HA configurations (unilateral right, unilateral left, or bilateral). Finally, the SPiN test itself was specifically developed for monaural presentation through headphones [42], further supporting the methodological approach used in this study.

#### 2.3.2. Mediator: Perceived Benefit

To evaluate the perceived benefit with HAs, question 2 from the same IOI-HAs instrument [45] was used: “*Piense en una situación donde usted realmente hubiera querido escuchar mejor antes de obtener su(s) audífono(s) actual(es). En las últimas dos semanas, ¿cuánto le ha ayudado el (los) audífono(s) en esa situación?*” [*Think about the situation where you most wanted to hear better, before you got your present hearing aid*(s). Over the past two weeks, how much *has the hearing aid helped in that situation?*]”. On an ordinal scale, the possible answers ranged from “*no ayudó (helped not at all)*”, “*ayudó poco (helped slightly)*”, “*ayudó moderadamente (helped moderately)*”, “*ayudó bastante (helped quite a lot)*”, “*ayudó mucho (helped very much)*”. The answers were coded from 5 points (i.e., *helped very much*) to 1 point (i.e., *helped not at all*).

#### 2.3.3. Outcome: Hearing Aid Use

To evaluate HA use, question 1 of the Spanish version of the IOI-HAs [45] was used: “*Piense cuanto ha utilizado usted su(s) audífono(s) actual(es) en las últimas dos semanas. En un día común*, *¿cuántas horas ha usado usted el(los) audífono(s)? [Think about how much you used your present hearing aid(s) over the past two weeks. On an average day, how many hours did you use the hearing aid(s)?]*”. Responses were measured on an ordinal scale ranging from *“ninguna (none)”*, *“menos de una hora al día (less than 1 h a day)”*, *“de 1 a 4 horas al día (1 to 4 h a day)”*, *“de 4 a 8 horas al día (4 to 8 h a day)”*, *to “más de 8 horas al día (more than 8 h a day)”*. The answers were coded from 5 points (i.e., more than 8 h a day) to 1 point (i.e., none).

### 2.4. Covariables

Sociodemographic information, health background, and other audiological variables were also collected to characterize the sample and control for potential confounding variables. The sociodemographic information included age, sex, years of formal education, monthly income, and number of cohabitants. Health background included general health perception, assessed with the following question: *“En general, ¿cómo describiría su salud? [In general, how would you describe your health?]”, with response options ranging from “mala (bad)”, “regular (alright)”, “buena (good)”, “muy buena (very good)”, to “excelente (excellent)”*; monthly spending on medication; the number of chronic diseases by asking about common health conditions in the national older adult population: hypertension (“¿*Alguna vez un doctor o enfermera le ha dicho que tiene la presión alta, es decir, hipertensión? [Has a doctor or nurse ever told you that you have high blood pressure, that is, hypertension?]*”), diabetes (“¿*Alguna vez un doctor o enfermera le ha dicho que tiene diabetes, es decir, niveles altos de azúcar en la sangre? [Has a doctor or nurse ever told you that you have diabetes, that is, high blood sugar levels?]*”), osteoarticular problems (“¿*Alguna vez un médico le ha dicho que tiene artritis, osteoporosis, arthrosis, o problemas en articulaciones? [Has a doctor ever told you that you have arthritis, osteoporosis, osteoarthritis, or joint problems?]*”), and cardiac problems (“*¿Alguna vez un doctor o enfermera le ha dicho que ha tenido un ataque al corazón, una enfermedad coronaria, angina, o insuficiencia cardíaca? [Has a doctor or nurse ever told you that you have had a heart attack, coronary artery disease, angina, or heart failure?]*”).

All this information was collected using questions from the Chilean National Survey of Dependency in Older Adults (ENADEAM in Spanish) [46], an instrument previously utilized in similar studies [22,23,41,47,48]. Additionally, the risk of depression was assessed through the 15-item Yesavage Geriatric Depression Scale (GDS-15) [49]. This scale comprises 15 dichotomous (yes/no) questions designed to evaluate various dimensions of mental health, including mood, life satisfaction, energy levels, and anxiety. Higher scores on the GDS indicate a greater likelihood of depression.

Audiological variables included the PTA for frequencies 500, 1000, 2000, and 4000 Hz, HA implementation laterality, months of experience using HAs, and self-efficacy with HAs. The PTA, HA implementation laterality, and months of experience were obtained from clinical records. For the PTA, the record of the latest audiometric exam was considered. No subject had an audiometric exam older than one year. Self-efficacy was measured using the basic handling (7 items) and advanced handling (5 items) subscales from the Spanish version of the Measure of Audiologic Rehabilitation Self-Efficacy for Hearing Aids (S-MARS-HA) [47]. These subscales assess the individual’s confidence in performing different tasks related to HA use. The S-MARS-HA responses were measured on a continuous scale from 0% (unable to perform the task) to 100% (completely confident in performing the task).

Additionally, although all the participants had a normal MMSE, the Addenbrooke’s Cognitive Examination Revised (ACE-R) was used to provide a more detailed cognitive profile. All the questionnaires and instruments that needed to be completed directly by the participants were provided in printed format, with large, easy-to-read fonts. If necessary, an evaluator read the questions and their possible answers aloud, after which the participants responded verbally or indicated their response by pointing with their hand.

### 2.5. Statistical Analysis

The following variables were analyzed as categorical variables: HA use, perceived benefit with HAs, self-perception of health status, sex, and HA implementation laterality. Descriptive statistics for these variables included absolute and relative frequencies. The variables analyzed as numerical variables were SPiN, age, years of education, monthly income, number of cohabitants, number of chronic diseases, monthly spending on medication, risk of depression, months of experience using HAs, self-efficacy with HAs, cognitive profile, and PTA. Descriptive statistics were reported as absolute and relative frequencies for categorical variables, and median (p50) with interquartile range (p25–p75) for numerical variables. The Mann–Whitney U test was used for comparisons between the ears for PTA, SPiN, and experience using HAs.

To identify potential confounders related to HA use and perceived benefit with HAs, univariate ordinal regression models were estimated, and odds ratios (OR) were obtained as measures of association. Variables that showed a statistically significant association (*p* < 0.1) were included in a multivariate ordinal generalized structural equation model (GSEM). This GSEM consisted of two multivariate ordinal regression models: one for HA use and one for perceived benefit with HAs. These models were integrated into the GSEM framework to evaluate an indirect or mediated effect between SPiN performance and HA use, with perceived benefit as the mediating factor. Additionally, the variables of age, PTA, and cognitive status were incorporated into this model because, according to the literature, they are associated with both SPiN performance [31] and HA use and perceived benefit with HAs [25] and thus were controlled for in the analysis.

**Figure 1:**
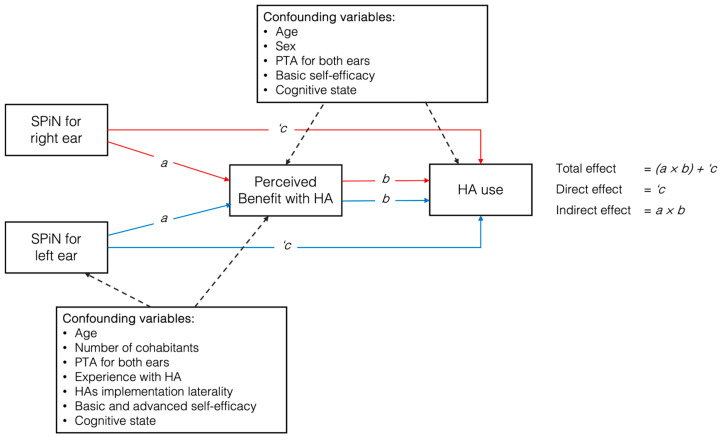
Proposed model of variable relationships between speech perception in noise (SPiN) and hearing aid use, mediated by perceived benefit, with covariates included for both ears. Note: HAs = hearing aids; PTA = pure tone average.

The mediation analysis included a direct path from SPiN (predictor) to HA use (outcome), as well as an indirect path from SPiN to perceived benefit with HAs (mediator), which then led to HA use (outcome). The total effect of SPiN on HA use was estimated by summing the direct and indirect effects. These estimations were conducted independently for the SPiN performance of each ear. The details of the model can be seen in Figure 1.

In the multivariate GSEM, the ordinal family and logit link were used, and the standard error was adjusted for clustering. An indirect (mediated) effect was considered present if the non-linear combination of the estimators—that is, the product of the path coefficient from SPiN performance to perceived benefit with HAs, and the path coefficient from perceived benefit with HAs to HA use—was statistically significant (*p* < 0.05) [46]. This procedure was conducted separately for each ear. The mediation model was carried out to avoid the presence of overadjustment bias in the total effect when the predictor and mediator variables are included in the multivariate ordinal regression model. Additionally, the mediated model allows for the decomposition of direct and indirect effects.

Effect sizes were reported as odds ratios with corresponding confidence intervals for both the direct and indirect pathways, as well as for the predictor–mediator and mediator–outcome pathways, following the recommendations outlined in the Guideline for Reporting Mediation Analyses of Randomized Trials and Observational Studies [50]. The guideline also indicates that reporting the proportion mediated is not always advisable, as there can be considerable uncertainty around it, especially in small samples. Thus, the focus should be on the indirect and direct effects. Additionally, VanderWeele [51] noted that the proportion mediated tends to be unstable and imprecise in small samples. In this study, given the limitations in sample size, the proportion mediated was not reported.

In addition, to conduct a sensitivity analysis of the results, the mediation model was re-estimated using the structural equation model (SEM) with partial least squares (PLS), considering the same variables used in the multivariate ordinal GSEM model. According to Venturini and Mehmetoglu [52], the use of this methodology is recommended for small sample sizes. The Monte Carlo method with 50,000 simulations was used to estimate 95% confidence intervals (95% CI). All the analyses were conducted using STATA v18 software. The “gsem” command of this software was used for GSEM estimation [53].

## 3. Results

### 3.1. Sample Description

Of the 114 participants, 57.98% (n = 66) were women. The median age was 79 years (p25–p75 = 73–85). A total of 55.75% (n = 63) of the participants used an HA only in the right ear. No significant differences were observed between the ears for PTA (*p* = 0.406), SPiN performance (*p* = 0.085), or months of experience with HA use (*p* = 0.156). The characteristics of the sample can be found in Table 1.

### 3.2. SPiN Performance

**Figure 2:**
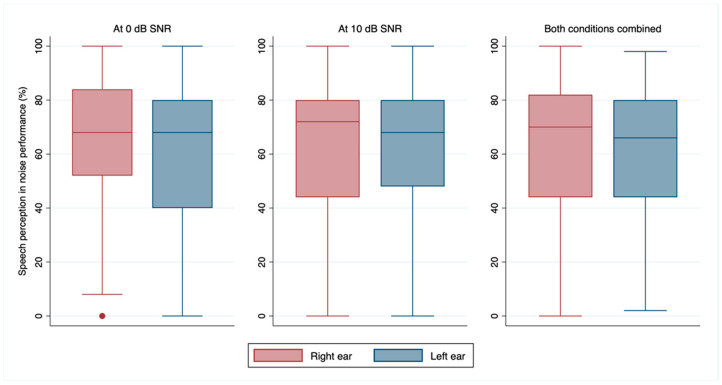
Median and 25th to 75th percentile of speech perception in noise performance at 0 dB and 10 dB SNR, and for both conditions combined for the left and right ears. Note: SNR = signal-to-noise ratio.

In the right ear, the median performance at 10 dB SNR was 72% (p25–p75 = 44–80), while at 0 dB SNR, it was 68% (p25–p75 = 52–84). The median performance of the left ear at 10 dB SNR was 68% (p25–p75 = 48–80), while at 0 dB SNR, it was 68% (p25–p75 = 40–80). The median combined performance was 70% (p25–p75 = 44–82) for the right ear and 66% (p25–p75 = 44–80) for the left ear. The detailed distribution of these performances can be seen in Figure 2.

### 3.3. Perceived Benefit with HAs and HA Use

Regarding question 2 of the IOI-HAs, perceived benefit with HAs, the most frequent responses were “helped quite a lot”, with 42.11% (n = 48), followed by “helped very much”, with 25.44% (n = 29), and “helped moderately”, with 13.16% (n = 15). The most frequent responses for question 1 of the IOI-HAs regarding HA use were “use for more than 8 h a day”, with 47.37% (n = 54), “1 to 4 h a day”, with 19.30% (n = 22), and “4 to 8 h a day”, with 15.79% (n = 18). The details of these and other responses can be seen in Table 2.

### 3.4. Variables Related to Outcome and Mediator

The results of the univariate ordinal regression models can be found in Table 3. Being female was negatively associated with the odds of advancing one category in HA use compared to being male (OR = 0.51, 95% CI 0.26–1.02; *p* = 0.056). Additionally, a positive association with HA use was observed both for each 10-point increase in the basic handling subscale of HA self-efficacy (OR = 1.16, 95% CI 0.97–1.39; *p* = 0.097) and for each category increase in perceived benefit with HAs (OR = 2.13, 95% CI 1.56–2.90; *p* < 0.001). Thus, greater self-efficacy and higher perceived *benefit were positively associated with increased HA use.*

In relation to perceived benefit with HAs, a negative association was observed with age, meaning that each additional year of age was associated with lower odds of reporting increased perceived benefit (OR = 0.93; 95% CI 0.89–0.98; p = 0.006). Conversely, perceived benefit increased significantly with each additional cohabitant in the household (OR = 1.51; 95% CI 1.09–2.08; *p* = 0.013) when using bilateral HAs compared to unilateral right-ear use (OR = 2.88; 95% CI 1.07–7.78; *p* = 0.037) and with each additional month of HA experience (OR = 1.01; 95% CI 1.00–1.01; *p* = 0.046). Higher self-efficacy scores in both basic (OR = 1.36; 95% CI 1.13–1.64; *p* = 0.001) and advanced handling of HAs (OR = 1.43; 95% CI 1.21–1.70; *p* < 0.001) also correlated positively with increased perceived benefit. Additionally, better SPiN performance in both the right ear (OR = 1.08; 95% CI 1.02–1.14; *p* = 0.008) and the left ear (OR = 1.19; 95% CI 1.11–1.27; *p* < 0.001) was associated with greater perceived benefit.

Regarding multivariate ordinal regression models, as the main outcome, HA use was solely and positively associated with perceived benefit with HAs (OR = 2.44; 95% CI 1.47–4.03; *p* = 0.001). This result corroborates that the effect of SPiN performance on HA use is primarily mediated, confirming the presence of overadjustment bias in the total effect when both the predictor and mediator variables are included in the multivariate ordinal regression model. In relation to perceived benefit with HAs, it was negatively associated with age (OR = 0.93; 95% CI 0.87–0.99; *p* = 0.022) and positively associated with SPiN for the left ear (OR = 1.29; 95% CI 1.09–1.48; *p* = 0.001). The rest of the variables that were significantly associated with both HA use and perceived benefit with HAs in the univariate models ceased to be significant.

### 3.5. Mediation Analysis

**Figure 3:**
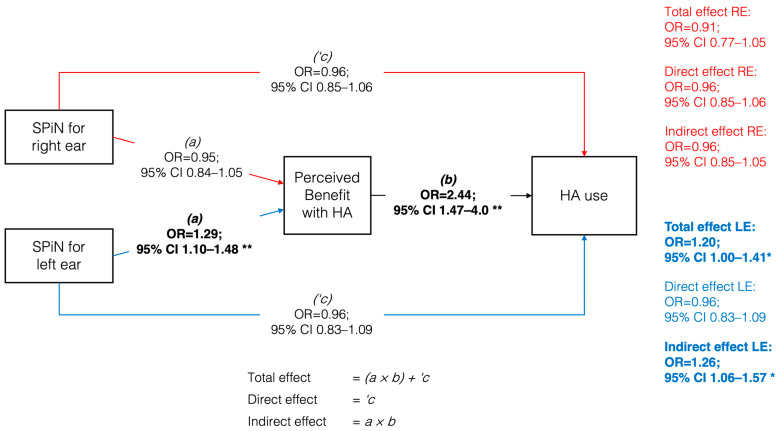
Generalized structural equation model between speech perception in noise (SPiN) for the right and left ear and hearing aids use, mediated by perceived benefit. Odds ratios expressed per 4-point change on SPiN. Note: RE = right ear; LE = left ear; OR = odds ratio; CI = confidence interval. * *p* < 0.05; ** *p* < 0.01. Bolded estimates indicate statistically significant effects.

Figure 3 presents the mediation model with its respective coefficients. SPiN performance in the right ear did not present a significant total effect (OR = 0.91; 95% CI 0.77–1.05; *p* = 0.228) on HA use. In the left ear, a significant indirect effect (OR = 1.26; 95% CI 1.06–1.57; *p* = 0.019) and a significant total effect (OR = 1.20; 95% CI 1.00–1.41; *p* = 0.041) were observed but not a direct effect (OR = 0.96; 95% CI 0.83–1.09; *p* = 0.563). These findings were replicated through a sensitivity analysis using a SEM model with partial least squares, revealing a positive and significant indirect effect of the SPiN path on the left ear on HA use, through perceived benefit (*p* < 0.001). Similarly, the results for the right ear remained consistent, with SPiN showing no significant direct effect with HA use (*p* = 0.095), aligning with the main results (see Table S1). Thus, participants with better SPiN performance in the left ear had higher odds of advancing in perceived benefit categories, which translated into higher odds of advancing in HA use categories.

Following the recommendations of the Guideline for Reporting Mediation Analyses of Randomized Trials and Observational Studies [50], the proportion mediated was not reported. However, the findings suggest that the observed effect is primarily mediated. This is supported by the fact that once the mediator was included in the multivariate ordinal regression model, the effect of SPiN was no longer statistically significant, indicating that the direct pathway did not reach statistical significance and that the observed effect is primarily attributable to the indirect pathway.

## 4. Discussion

### 4.1. Mediated Association Between Unaided SPiN and HA Use

The primary objective of the present study was to evaluate the direct and indirect associations, through perceived benefit with HAs, between unaided SPiN performance and HA use. In our mediation analysis, SPiN performance was positively associated with perceived benefit, which, in turn, contributed to increased odds of HA use. Ear-specific effects were observed: whereas the SPiN performance in the right ear showed no significant total effect, a significant total effect was observed for the left ear, which was mainly mediated by perceived benefit (significant indirect effect).

The association between unaided SPiN performance and HA use, mediated through perceived benefit, had not been previously studied. However, prior research has demonstrated a relationship between SPiN performance and perceived benefit [34,36]. Mendel [36] examined 21 HA users aged 33 to 75 years, 17 of whom were bilateral users, with HA experience ranging from 6 months to 6 years (mean = 1.7 years). The study found a significant association between unaided SPiN performance and benefit-related questions from the HAPI questionnaire, specifically those assessing the perception of environmental sounds in quiet and noisy conditions, as well as conversations with both familiar and unfamiliar talkers. SPiN performance was measured using the R-SPIN, HINT, and QuickSIN, all presented in a free-field setup. However, Mendel [36] did not investigate potential associations between SPiN performance and HA use.

Walden and Walden [34] studied 50 adults aged 49 to 94 years, 39 of whom were bilateral HA users, with experience ranging from 2 months to 20 years (mean = 5.3 years). They found that better performance on the unaided QuickSIN test was associated with higher scores on the HAUS—that is, better perceived benefit. This scale estimates the patient’s overall perception of the usefulness of their HA in daily life on a scale from 1 to 100. A score of 1 indicates, “My hearing aid(s) are of no use to me”, while a score of 100 indicates, “My hearing aid(s) are so useful that they meet every need I have for them”. Similar to Mendel’s [36] study, the association between SPiN performance and HA use was not examined.

Similar to Mendel’s [36] and Walden and Walden’s [34] works, the participants in the present study had a wide range of experience using HA. Since this variable was significantly associated with perceived benefit with HAs in the univariate model, it was controlled for in the multivariate GSEM model. The association between SPiN performance and perceived benefit with HAs, as well as the indirect effect of SPiN on HA use, remained significant in the multivariate GSEM model. This finding suggests that unaided SPiN measures could serve as a reliable predictor of these HA outcomes in both new and experienced HA users, unlike unaided speech perception measures in quiet conditions, which have demonstrated significant associations only among new HA users [20,27].

In relation to HA experience, the recent systematic review by Mothemela et al. [25] identified only one study examining its association with HA use, and that study reported no significant relationship. Moreover, no studies were found that evaluated the link between HA experience and perceived benefit. In our study, longer-term HA experience—measured as the number of months using the device—was significantly associated with greater perceived benefit in the univariate analysis. This finding suggests that users may perceive more benefit as their experience with the device increases, potentially due to improved adaptation, greater familiarity with its features, or more effective integration into everyday communication. However, this relationship may also reflect reverse causality, whereby individuals who perceive greater benefit are more likely to continue using their HA consistently, thereby accumulating more months of use. Future longitudinal studies are needed to clarify whether extended HA use enhances perceived benefit or, conversely, whether perceived benefit encourages continued device use over time.

In contrast to the findings of this study, and those reported by Mendel [36] and Walden and Walden [34], Grunditz and Magnusson [54] did not find an association between SPiN performance and perceived benefit with HAs. These authors studied 102 subjects aged 24 to 91 years, 96 of whom were bilateral HA users, and all of whom were new users with 12 to 16 weeks of experience. The study employed a free-field SPiN test using monosyllabic words presented in the presence of an artificial noise signal. This noise had the same modulation properties as natural speech but was completely unintelligible and exhibited a speech-like spectrum, with an SNR of +6 dB. The SPiN performance in the unaided condition was not associated with Item 2 (perceived benefit with HAs) of the IOI-HAs. However, it was significantly associated with Item 3 (residual activity restriction), Item 5 (residual participation restriction), and Item 6 (impact on others), as well as with the total IOI-HAs score.

The discrepancy between the findings of Grunditz and Magnusson [54] and those of the present study, as well as Mendel [36] and Walden and Walden [34], may be attributed to differences in the type of SPiN test used. Grunditz and Magnusson used a speech recognition in noise test with a fixed, favorable SNR of +6 dB, which likely reduced the overall difficulty of the task. In contrast, Mendel [36] and Walden and Walden [34] utilized tests designed to determine the speech reception threshold, reaching more challenging SNRs—QuickSIN, for example, includes conditions as low as 0 dB SNR, and HINT is an adaptive test that adjusts to pinpoint the SNR at which 50% of the speech is correctly understood. In the present study, we used a SPiN test with two conditions—one at +10 dB and another at 0 dB—thus incorporating a more challenging SNR condition. More challenging SNR conditions may have greater ecological validity, given that real-world SNRs are rarely as favorable. In contrast, the relatively easier SNR condition used by Grunditz and Magnusson may have limited the test’s sensitivity to variations in perceived benefit, contributing to the absence of an association between SPiN performance and perceived benefit with HAs.

On the other hand, the relationship between perceived benefit with HAs and HA use has also been demonstrated in previous studies [20,21]. In the study by Houmøller et al. [20], two groups were examined: 1090 new HA users and 417 experienced users (with more than 4 years of experience). In both groups, daily hours of HA use were significantly associated with Factor 1 of the IOI-HAs, which includes a question on perceived benefit. Similarly, Wang et al. [21] found that daily HA use was associated with better scores on Factor 1 of the IOI-HAs in a sample of 235 adult HA users with usage experience ranging from 3 months to 20 years.

Additionally, a lack of perceived benefit with HAs has been identified as one of the main reasons for discontinuation of HA use among older adults [22,23,24]. In a first retrospective cohort study, Fuentes-López et al. [22] investigated 355 older adult HA users. At 30 months post-fitting, the cumulative incidence of HA discontinuation was 21.7%, and among those who discontinued, 18.2% reported little or no perceived benefit. In a subsequent study with similar characteristics, Fuentes-López et al. [23] examined 455 older adult HA users with at least one year of HA use. Within this cohort, 18% of the participants had discontinued use of their devices; notably, among these individuals, 26.3% reported little or no perceived benefit. In both studies, the lack of perceived benefit was the most common reason for HA abandonment.

Iwahashi et al. [24] investigated two groups of adult HA users, subjects who had received their devices one year earlier and were invited to a follow-up and control session. The first group comprised 200 subjects who attended the session, while the second group consisted of 108 who did not. In the first group, 13% discontinued HA use, and among those, 17% cited a lack of perceived benefit—the second most frequent reason for discontinuation. In the second group, 30.6% discontinued use, with 13.2% reporting a lack of perceived benefit, making it the third most common cause of HA discontinuation.

An unexpected finding in the present study was that the mediated relationship between unaided SPiN performance and HA use differed by ear. Specifically, only the SPiN performance in the left ear was significantly associated with HA use, even after adjusting for HA configuration (i.e., whether subjects used right unilateral, left unilateral, or bilateral adaptations). Previous studies examining the association between SPiN performance and perceived benefit with HAs [34,36,54], and between SPiN performance and HA use [54] employed psychoacoustic tests presented binaurally in a free-field setup. In contrast, the SPiN test used in the present study was administered monaurally via headphones, allowing for independent scoring for each ear.

Some studies have proposed that the auditory pathway responsible for left ear input may be more affected by aging than the right ear pathway, resulting in earlier and more pronounced declines in speech processing [55]. This hypothesis is supported by psychoacoustic [56,57] and electrophysiological studies [58,59,60], which show a weaker left ear for speech processing among older adults in both unaided and aided conditions [61]. Furthermore, research conducted on older adults has found that left ear SPiN scores tend to be lower than those for the right ear, particularly in the presence of background noise [61,62,63]. These interaural processing differences may compromise older adults’ ability to use binaural cues for analyzing the spatial characteristics of complex acoustic environments [64], potentially leading to binaural interference. Such interference could reduce the actual benefits provided by HA and may ultimately contribute to decreased HA use [65,66].

Our study showed no significant differences in SPiN performance between the ears, suggesting reduced interaural asymmetry. We hypothesize that this balanced auditory processing led to improved binaural integration, which, in turn, enhanced the perceived benefit of HAs and ultimately increased their use, even when the device was not specifically used in the left ear. However, with our previous data, we cannot confirm this hypothesis because SPiN was not assessed before HA fitting, thus it is not possible to determine whether the similar auditory processing between the ears was a result of HA utilization or reflects a pre-existing condition [67].

Although only left ear SPiN performance was significantly associated with HA use in the mediation analysis, more than half of the participants were fitted with an HA in the right ear only. In addition, within the GES program, when only one device is provided, it is commonly placed in the right ear for practical reasons, as most users are right-handed [68]. Consequently, the aided ear is not necessarily the one with better residual hearing or superior SPiN performance. Interestingly, bilateral HA fitting was significantly associated with higher perceived benefit in our univariate model. This is a noteworthy finding, as the recent systematic review by Mothemela et al. [25] did not identify any studies that explicitly examined the association between HA laterality and perceived benefit. While the review reported mixed results regarding bilateral fitting and HA use, the relationship with perceived benefit remains largely unexplored in the literature. Nonetheless, in our analyses, HA laterality was included as a covariate and adjusted for in the multivariate mediation model. As such, the indirect association between left ear SPiN performance and HA use—mediated through perceived benefit—remained statistically independent of the laterality of HA implementation.

### 4.2. Direct Association Between Unaided SPiN and Hearing Aid Use

The secondary objective of the present study was to assess the direct association between unaided SPiN performance and HA use. In this analysis, the SPiN performance in neither ear showed a direct association with HA use. This result aligns with the findings of Grunditz and Magnusson [54], who found no association between their SPiN test and HA use (as measured by question 1 of the IOI-HAs), although they did observe associations between unaided SPiN performance and Factor 2 items (residual activity limitations, residual participation restriction, and impact on others), as well as the total IOI-HAs score. These results suggest that SPiN performance may be indirectly related to HA use via its influence on other outcome variables, consistent with Houmøller et al. [20], who reported that higher Factor 2 scores were linked to increased daily HA use.

The lack of a direct association between unaided SPiN performance and HA use observed in the present study may be attributable to the underlying mechanisms influencing HA utilization. It is likely that decreases in HA use occur predominantly when individuals explicitly perceive an insufficient benefit or encounter substantial communication challenges, rather than being directly driven by their objective SPiN performance. In other words, lower unaided SPiN performance alone might not lead to decreased HA use unless it translates into noticeable dissatisfaction or significant limitations in everyday listening situations. Future studies could explore specific SPiN thresholds at which the perceived benefit meaningfully decreases, thus directly impacting HA use. Identifying these thresholds would enable clinicians to proactively manage perceived communication difficulties and potentially prevent HA abandonment through tailored interventions and individualized counseling strategies.

### 4.3. Clinical Implications

The present findings have relevant implications for audiological assessment and rehabilitation. First, the observed indirect association between unaided SPiN performance and HA use—mediated through perceived benefit—suggests that SPiN measures can provide clinically meaningful information about potential HA outcomes. This information could be used to support realistic expectation management regarding the perceived benefit patients may experience with HA. Effectively managing expectations may help prevent disappointment and disengagement, particularly by addressing patients’ attitudes, beliefs, and motivation—factors that have been associated with HA use and perceived benefit [25,69].

The potential clinical utility of SPiN testing lies in its ecological validity—that is, its ability to reflect the complex auditory demands of real-world listening environments [29]. Binaural SPiN tests are particularly valuable in this regard, as they account for binaural integration and spatial hearing—fundamental skills for speech perception in noise [70,71], which are known to decline with age [72]. However, monaural SPiN tests also offer important clinical information by isolating ear-specific performance. In the present study, the fact that the relationship between unaided SPiN and HA use was significant only for the left ear, despite adjusting for HA configuration, underscores the relevance of ear-specific assessment in older adults. Audiologists may benefit from combining monaural and binaural SPiN measures to obtain a more complete understanding of functional hearing and guide individualized rehabilitation.

Importantly, SPiN performance is not a static ability but a modifiable auditory function. Evidence from recent studies indicates that both monaural and binaural SPiN outcomes can improve through targeted auditory training programs [73]. These interventions may focus on bottom-up mechanisms, such as pitch, spatial, or temporal cue processing, as well as top-down cognitive functions, like working memory and attention. Patients with limited unaided SPiN abilities may benefit from structured training protocols aimed at improving these underlying mechanisms. Tailoring follow-up and rehabilitation strategies based on individual SPiN profiles may contribute to more effective, targeted, and responsive care.

### 4.4. Limitations

A major limitation of this study design is that it does not allow for a clear determination of the temporal relationship between SPiN performance and HA outcomes. In the review by Lavie et al. [67], some findings suggest that HA use may induce neuronal plasticity changes that improve unaided SPiN performance. Although the evidence has not been entirely consistent, it is possible that SPiN performance measured prior to HA fitting may be modified by subsequent HA use. In the present study, all the participants had already initiated HA use at the time of SPiN testing. The median duration of HA use was 24 months (interquartile range: 12–48 months), which indicates that participants had varying levels of auditory experience prior to SPiN assessment. While the results reported in the review by Lavie et al. suggest amplification-induced improvements in speech perception over time, these findings should be interpreted with caution, as the observed improvements were small, and the methodological quality of the studies was moderate. Future research using longitudinal designs is needed to better understand the directionality and potential causality of these relationships.

As a second limitation, it should be noted that HA use was measured via self-reporting using question 1 of the IOI-HAs. Some research has indicated that self-reports tend to overestimate actual HA use measured by data-logging [74,75]; however, one previous study has shown minimal discrepancies between self-reported HA usage and data-logging, with an average overestimation of only 1.2 h [74]. Additionally, the IOI-HAs is a standardized and widely used tool for assessing HA use in the literature [76], which facilitates comparisons with other studies. Nonetheless, these estimates may be influenced by the structure of the response options. The IOI-HAs includes broad categorical intervals (e.g., “1 to 4 h,” “4 to 8 h”) that likely minimize the impact of small reporting discrepancies in moderate-use ranges. However, ceiling effects have been reported for IOI-HAs question 1, with a large proportion of users selecting “more than 8 h per day” [74]. In our sample, 47.37% chose this option, reducing variability at the upper end of the scale. This may attenuate associations with other variables and underestimate effect sizes or indirect effects in mediation models. Over-reporting also appears more pronounced in the lowest usage categories. Solheim and Hickson [75] found that participants who did not use their HA at all (per data-logging) reported nearly two hours of daily use. Such misreporting may increase misclassification risk in models examining minimal use. These potential biases should be considered when interpreting associations involving self-reported HA use, and future studies would benefit from including both subjective and objective usage measures.

Despite the limitations of the present study, the use of a mediation model represents a strength, as it provides a deeper understanding of the relationships between unaided SPiN performance, perceived benefit, and HA use. Unlike previous studies that analyzed these variables independently, mediation analysis identified indirect pathways, explicitly highlighting the mediating role of perceived benefit with HAs. Furthermore, mediation analysis helps mitigate the risk of overadjustment bias inherent in conventional multivariate models. For instance, SPiN performance in the left ear was significantly associated with perceived benefit but ceased to be significant in the multivariate ordinal regression model. This suggests that mediation analysis helps mitigate the risk of overadjustment bias inherent in conventional multivariate models. Thus, the conclusions would have been biased if a conventional analysis strategy based on multivariate regression models had been used.

## 5. Conclusions

In the present study, a mediated or indirect association was observed between unaided SPiN performance and HA use through perceived benefit with HAs. This association was independent of HA experience and bilateral or unilateral adaptations, yet it was observed only for the left ear. Additionally, no direct association between unaided SPiN performance and HA use was observed. These findings suggest that psychoacoustic measures of SPiN may serve as a useful tool for guiding the HA fitting process, providing counseling on realistic expectations for new HA users, and planning potential interventions.

## Figures and Tables

**Table 1:** Characteristics of the sample of Chilean older adults (n = 114) who were fitted with hearing aids in the public healthcare sector.

Variable	n (%) or p50 (p25–p75)
**Women in the sample**	66 (57.89)
**Age in years**	79 (73–85)
**Years of education**	6 (4–11)
**Number of cohabitants**	2 (2–3)
**Self-perception of state of health**	
Excellent	5 (4.39)
Very good	5 (4.39)
Good	37 (32.46)
Alright	49 (42.98)
Bad	18 (15.79)
**Number of chronic illnesses**	2 (1–3)
**Monthly income (in Chilean pesos)**	$180,000 ($140,000–220,000)
**Monthly spending on medication (in Chilean pesos)**	$15,000 ($0–30,000)
**Hearing aids implementation laterality**	
Only on right ear	63 (55.75)
Only on left ear	33 (29.20)
On both ears	17 (15.04)
**Months of experience using hearing aids**	24 (12–48)
**Pure tone average**	
Right ear	62.5 (52.50–72.50)
Left ear	61.3 (51.60–69.40)
**Self-efficacy with hearing aids (S-MARS-HA)**	
Basic handling scale	77.1 (60–88.60)
Advanced handling scale	70 (48–80)
**Depression (GDS)**	4 (2–7)
**Cognitive state (ACE-R)**	71.50 (59–80)

S-MARS-HA = Spanish version of Measure of Audiologic Rehabilitation Self-Efficacy for Hearing Aids; GDS = Yesavage Geriatric Depression Scale; ACE-R = Addenbrooke’s Cognitive Examination—Revised.

**Table 2:** Absolute frequency and percentage of responses for questions 1 and 2 of the Spanish version of the IOI-HAs questionnaire.

Answer Category	Question 2 of IOI-HAs ^b^ n (%)	Question 1 of IOI-HAs ^a^ n (%)
1	12 (10.53)	16 (14.04)
2	10 (8.77)	4 (3.51)
3	15 (13.16)	22 (19.30)
4	48 (42.11)	18 (15.79)
5	29 (25.44)	54 (47.37)
Total	114 (100)	114 (100)

IOI-HAs = International Outcome Inventory for Hearing Aids. Statistically significant effects are marked in bold. ^a^ “Think about how much you used your present hearing aid(s) over the past two weeks. On an average day, how many hours did you use the hearing aids?” with possible answers 1: None, 2: Less than 1 h/day, 3: 1–4 h/day, 4: 4–8 h/day, 5: More than 8 h/day. ^b^ “Think about the situation where you most wanted to hear better, before you got your present hearing aid(s). Over the past two weeks, how much has the hearing aid helped in that situation?” with possible answers 1: Helped not at all, 2: Helped slightly, 3: Helped moderately, 4: Helped quite a lot, 5: Helped very much.

**Table 3:** Univariate ordinal regressions for HA use and perceived benefit with HAs.

Variables	Univariate Model for HAs Use OR (95% CI)	*p*-Value	Univariate Model for Perceived Benefit OR (95% CI)	*p*-Value
**Being a woman**	**0.51 (0.26–1.02)**	**0.056**	1.13 (0.57–2.21)	0.731
**Age in years**	1.01 (0.97–1.06)	0.581	**0.93 (0.89–0.98)**	**0.006**
**Years of education**	0.97 (0.89–1.05)	0.428	1.01 (0.93–1.09)	0.885
**Number of cohabitants**	1.01 (0.75–1.37)	0.952	**1.51 (1.09–2.08)**	**0.013**
**Self-perception of state of health**				
Excellent	*Reference*	-	*Reference*	-
Very good	0.35 (0.02–5.03)	0.436	1.12 (0.13–9.47)	0.917
Good	0.48 (0.05–4.87)	0.534	1.36 (0.26–7.11)	0.719
Alright	0.19 (0.02–1.90)	0.158	0.79 (0.16–3.99)	0.778
Bad	0.19 (0.02–2.09)	0.175	0.42 (0.07–2.38)	0.327
**Number of chronic illnesses**	0.83 (0.52–1.30)	0.411	0.85 (0.54–1.35)	0.490
**Monthly income (in Chilean pesos)**	0.99 (0.99–1.00)	0.570	1.00 (0.99–1.00)	0.938
**Months of experience with hearing aids**	1.00 (1.00–1.01)	0.660	**1.01 (1.00–1.01)**	**0.046**
**Monthly spending on medication (in Chilean pesos)**	0.99 (0.99–1.00)	0.789	0.99 (0.99–1.00)	0.616
**Hearing aids implementation laterality**				
Only on right ear	*Reference*	-	*Reference*	-
Only on left ear	0.63 (0.29–1.40)	0.260	1.90 (0.85–4.22)	0.117
On both ears	2.11 (0.77–5.76)	0.147	**2.88 (1.07–7.78)**	**0.037**
**Pure tone average (frequencies 500, 1000, 2000, and 4000 Hz)**				
Right ear	1.02 (0.99–1.04)	0.183	1.02 (1.00–1.04)	0.117
Left ear	1.00 (0.97–1.02)	0.910	0.99 (0.96–1.02)	0.447
**Self-efficacy with hearing aids (S-MARS-HA)**				
Basic handling subscale ^a^	**1.16 (0.97–1.39)**	**0.097**	**1.36 (1.13–1.64)**	**0.001**
Advanced handling subscale ^a^	1.01 (1.00–1.03)	0.185	**1.43 (1.21–1.70)**	**<0.001**
**Depression (Yesavage Geriatric Depression Scale)**	1.07 (0.96–1.18)	0.219	0.95 (0.86–1.05)	0.303
**Speech perception in noise test (SPIN test)**				
On the right ear ^b^	0.98 (0.93–1.04)	0.542	**1.08 (1.02–1.14)**	**0.008**
On the left ear ^b^	1.02 (0.96–1.08)	0.570	**1.19 (1.11–1.27)**	**<0.001**
**Cognitive state (Addenbrooke’s Cognitive Examination—Revised)**	0.99 (0.97–1.02)	0.485	1.00 (0.98–1.02)	0.966
**Perceived benefit** (Question N°2 from the IOI-HAs questionnaire)	**2.13 (1.56–2.90)**	**<0.001**	-	-

OR = odds ratio; CI = confidence interval; IOI-HAs: International Outcome Inventory for Hearing Aids; S-MARS-HA= Spanish version of Measure of Audiologic Rehabilitation Self-Efficacy for Hearing Aids. ^a^ Change expressed per 10-point increase. ^b^ Change expressed per 4-point increase. Note: Statistically significant variables with *p* < 0.1 are presented in bold.

## Data Availability

The dataset used and analyzed during the current study is available from the corresponding author upon reasonable request.

## Supplementary Materials

The following supporting information can be downloaded at: https://www.mdpi.com/article/10.3390/audiolres15030050/s1, Table S1: Estimation of the indirect effect of SPIN on HA use mediated by the perceived benefit of using a HA ^a^.

## Abbreviations

The following abbreviations are used in this manuscript:

SPiN	Speech perception in noise
HA	Hearing aid

## References

[1] World Health Organization (WHO) Deafness and Hearing Loss. https://www.who.int/news-room/fact-sheets/detail/deafness-and-hearing-loss.

[2] Kassebaum N.J., Arora M., Barber R.M., Bhutta Z.A., Brown J., Carter A., Casey D.C., Charlson F.J., Coates M.M., Coggeshall M. (2016). Global, regional, and national disability-adjusted life-years (DALYs) for 315 diseases and injuries and healthy life expectancy (HALE), 1990–2015: A systematic analysis for the Global Burden of Disease Study 2015. Lancet.

[3] Shukla A., Harper M., Pedersen E., Goman A., Suen J.J., Price C., Applebaum J., Hoyer M., Lin F.R., Reed N.S. (2020). Hearing Loss, Loneliness, and Social Isolation: A Systematic Review. Otolaryngol. Head. Neck Surg..

[4] Lawrence B.J., Jayakody D.M.P., Bennett R.J., Eikelboom R.H., Gasson N., Friedland P.L. (2020). Hearing Loss and Depression in Older Adults: A Systematic Review and Meta-analysis. Gerontologist.

[5] Jiam N.T.L., Li C., Agrawal Y. (2016). Hearing loss and falls: A systematic review and meta-analysis. Laryngoscope.

[6] Loughrey D.G., Kelly M.E., Kelley G.A., Brennan S., Lawlor B.A. (2018). Association of Age-Related Hearing Loss With Cognitive Function, Cognitive Impairment, and Dementia: A Systematic Review and Meta-analysis. JAMA Otolaryngol. Head Neck Surg..

[7] Yeo B.S.Y., Song H.J.J.M.D., Toh E.M.S., Ng L.S., Ho C.S.H., Ho R., Merchant R.A., Tan B.K.J., Loh W.S. (2023). Association of Hearing Aids and Cochlear Implants with Cognitive Decline and Dementia: A Systematic Review and Meta-analysis. JAMA Neurol..

[8] Hanratty B., Lawlor D. (2000). Effective management of the elderly hearing impaired—A review. J. Public. Health Med..

[9] Huang Q., Tang J. (2010). Age-related hearing loss or presbycusis. Eur. Arch. Oto-Rhino-Laryngol..

[10] Zheng Q., Xu Z., Li N., Wang Y., Zhang T., Jing J. (2024). Age-related hearing loss in older adults: Etiology and rehabilitation strategies. Front. Neurosci..

[11] Dornhoffer J.R., Meyer T.A., Dubno J.R., McRackan T.R. (2020). Assessment of Hearing Aid Benefit Using Patient-Reported Outcomes and Audiologic Measures. Audiol. Neurotol..

[12] Ferguson M.A., Kitterick P.T., Chong L.Y., Edmondson-Jones M., Barker F., Hoare D.J. (2017). Hearing aids for mild to moderate hearing loss in adults. Cochrane Database Syst. Rev..

[13] Lin F.R., Pike J.R., Albert M.S., Arnold M., Burgard S., Chisolm T., Couper D., Deal J.A., Goman A.M., Glynn N.W. (2023). Hearing intervention versus health education control to reduce cognitive decline in older adults with hearing loss in the USA (ACHIEVE): A multicentre, randomised controlled trial. Lancet.

[14] Dillon H., Day J., Bant S., Munro K.J. (2020). Adoption, use and non-use of hearing aids: A robust estimate based on Welsh national survey statistics. Int. J. Audiol..

[15] Aazh H., Prasher D., Nanchahal K., Moore B.C.J. (2015). Hearing aid use and its determinants in the UK National Health Service: A cross-sectional study at the Royal Surrey County Hospital. Int. J. Audiol..

[16] Cox R.M. (2003). Assessment of subjective outcome of hearing aid fitting: Getting the client’s point of view. Int. J. Audiol..

[17] Jilla A.M., Johnson C.E., Danhauer J.L., Anderson M., Smith J.N., Sullivan J.C., Sanchez K.R. (2020). Predictors of Hearing Aid Use in the Advanced Digital Era: An Investigation of Benefit, Satisfaction, and Self-Efficacy. J. Am. Acad. Audiol..

[18] Stark P., Hickson L. (2004). Outcomes of hearing aid fitting for older people with hearing impairment and their significant others. Int. J. Audiol..

[19] Cox R.M., Alexander G.C. (1992). Amplification and Aural Rehabilitation Maturation of Hearing Aid Benefit: Objective and Subjective Measurements. Ear Hear..

[20] Houmøller S.S., Wolff A., Möller S., Narne V.K., Narayanan S.K., Godballe C., Hougaard D.D., Loquet G., Gaihede M., Hammershøi D. (2022). Prediction of successful hearing aid treatment in first-time and experienced hearing aid users: Using the International Outcome Inventory for Hearing Aids. Int. J. Audiol..

[21] Wang X., Zheng Y., Liu Y., Lu J., Cui Z., Li Z. (2022). Effects of demographic, audiologic, and hearing-aid-related variables on the outcomes of using hearing aids. Eur. Arch. Oto-Rhino-Laryngol..

[22] Fuentes-López E., Fuente A., Valdivia G., Luna-Monsalve M. (2019). Effects of auditory and socio-demographic variables on discontinuation of hearing aid use among older adults with hearing loss fitted in the Chilean public health sector. BMC Geriatr..

[23] Fuentes-López E., Galaz-Mella J., Ayala S., De la Fuente C., Luna-Monsalve M., Nieman C., Marcotti A. (2024). Association be-tween the home-to-healthcare center distance and hearing aid abandonment among older adults. Front. Public. Health.

[24] Jardim I.d.S., Shirayama Y., Yuasa M., Bento R.F., Iwahashi J.H. (2015). Hearing aid use and adherence to treatment in a publicly-funded health service from the city of São Paulo, Brazil. Int. Arch. Otorhinolaryngol..

[25] Mothemela B., Manchaiah V., Mahomed-Asmail F., Knoetze M., Swanepoel D.W. (2024). Factors influencing hearing aid use, benefit and satisfaction in adults: A systematic review of the past decade. Int. J. Audiol..

[26] Hoppe U., Hocke T., Iro H. (2022). Age-Related Decline of Speech Perception. Front. Aging Neurosci..

[27] Chang Y.S., Choi J., Moon I.J., Hong S.H., Chung W.H., Cho Y.S. (2016). Factors associated with self-reported outcome in adaptation of hearing aid. Acta Otolaryngol..

[28] Wu X., Ren Y., Wang Q., Li B., Wu H., Huang Z., Wang X. (2019). Factors associated with the efficiency of hearing aids for patients with age-related hearing loss. Clin. Interv. Aging.

[29] Billings C.J., Olsen T.M., Charney L., Madsen B.M., Holmes C.E. (2024). Speech-in-Noise Testing: An Introduction for Audiologists. Semin. Hear..

[30] Weissgerber T., Müller C., Stöver T., Baumann U. (2022). Age Differences in Speech Perception in Noise and Sound Localization in Individuals With Subjective Normal Hearing. Front. Psychol..

[31] Kocabay A.P., Aslan F., Yüce D., Turkyilmaz D. (2022). Speech in Noise: Implications of Age, Hearing Loss, and Cognition. Folia Phoniatr. Logop..

[32] Heidari A., Moossavi A., Yadegari F., Bakhshi E., Ahadi M. (2018). Effects of age on speech-in-noise identification: Subjective ratings of hearing difficulties and encoding of fundamental frequency in older adults. J. Audiol. Otol..

[33] Lavie L., Banai K., Attias J., Karni A. (2014). How difficult is difficult? Speech perception in noise in the elderly hearing impaired. J. Basic. Clin. Physiol. Pharmacol..

[34] Walden T.C., Walden B.E. (2004). Predicting success with hearing aids in everyday living. J. Am. Acad. Audiol..

[35] Killion M.C., Niquette P.A., Gudmundsen G.I., Revit L.J., Banerjee S. (2004). Development of a quick speech-in-noise test for measuring signal-to-noise ratio loss in normal-hearing and hearing-impaired listeners. J. Acoust. Soc. Am..

[36] Mendel L.L. (2007). Objective and Subjective Hearing Aid Assessment Outcomes. Am. J. Audiol..

[37] Wilson R.H., McArdl R., Watt K.L., Smith S.L. (2012). The revised speech perception in noise test (R-SPIN) in a multiple signal-to-noise ratio paradigm. J. Am. Acad. Audiol..

[38] Nilsson M., Soli S.D., Sullivan J.A. (1994). Development of the Hearing in Noise Test for the measurement of speech reception thresholds in quiet and in noise. J. Acoust. Soc. Am..

[39] on Elm E., Altman D.G., Egger M., Pocock S.J., Gøtzsche P.C., Vandenbroucke J.P., Initiative S. (2007). The Strengthening the Reporting of Observational Studies in Epidemiology (STROBE) statement: Guidelines for reporting observational studies. Lancet.

[40] Quiroga P., Albala B.C., Klaasen G. (2004). Validación de un test de tamizaje para el diagnóstico de demencia asociada a edad, en Chile Validation of a screening test for age associated cognitive impairment, in Chile. Rev. Med. Chil..

[41] Fuentes-López E., Fuente A., Cardemil F., Valdivia G., Albala C. (2017). Prevalence and associated factors of hearing aid use among older adults in Chile. Int. J. Audiol..

[42] Fuente A., McPherson B. (2006). Auditory processing tests for Spanish-speaking adults: An initial study. Int. J. Audiol..

[43] Lankford J.E., Perrone D.C., Ma T.D., Thunder M.A. (1999). Ambient Noise Levels in Mobile Audiometric Testing Facilities: Compliance with Industry Standards. AAOHN J..

[44] British Society of Audiology (2019). Practice Guidance: The Acoustics of Sound Field Audiometry in Clinical Audiological Applications. www.thebsa.org.uk.

[45] Cox R.M., Stephens D., Kramer S.E. (2002). Translations of the International Outcome Inventory for Hearing Aids (IOI-HA): Traducciones del Inventario Internacional de Resultados para Auxiliares Auditivos (IOI-HA). Int. J. Audiol..

[46] Servicio Nacional del Adulto Mayor Estudio Nacional de Dependencia en las Personas Mayores. https://www.senama.gob.cl/storage/docs/Dependencia-Personas-Mayores-2009.pdf.

[47] Fuentes-López E., Fuente A., Valdivia G., Luna-Monsalve M. (2019). Does educational level predict hearing aid self-efficacy in experienced older adult hearing aid users from Latin America? Validation process of the Spanish version of the MARS-HA questionnaire. PLoS ONE.

[48] Fuentes-López E., Luna-Monsalve M., Silva-Letelier C., Marcotti A. (2024). Interaction effect of self-efficacy and joint problems on hearing aid abandonment among older adults. Int. J. Audiol..

[49] Gallardo-Peralta L.P., Rodríguez-Blázquez C., Ayala-García A., Forjaz M.J. (2020). Multi-ethnic validation of 15-item Geriatric Depression Scale in Chile. Psicol. Reflex. E Critica.

[50] Lee H., Cashin A.G., Lamb S.E., Hopewell S., Vansteelandt S., Vanderweele T.J., MacKinnon D.P., Mansell G., Collins G.S., Golub R.M. (2021). A Guideline for Reporting Mediation Analyses of Randomized Trials and Observational Studies: The AGReMA Statement. JAMA.

[51] VanderWeele T. (2015). Explanation in Causal Inference: Methods for Mediation and Interaction.

[52] Venturini S., Mehmetoglu M. (2019). Plssem: A stata package for structural equation modeling with partial least squares. J. Stat. Softw..

[53] Kenny D.A. (2017). MedPower: An Interactive Tool for the Estimation of Power in Tests of Mediation. https://davidakenny.shinyapps.io/MedPower/.

[54] Grunditz M., Magnusson L. (2013). Validation of a speech-in-noise test used for verification of hearing aid fitting. Hear. Balance Commun..

[55] Jerger J., Chmiel R., Allen J., Wilson A. (1994). Effects of Age and Gender on Dichotic Sentence Identification. Ear Hear..

[56] Roup C. (2011). Dichotic Word Recognition in Noise and the Right-Ear Advantage. J. Speech Lang. Hear. Res..

[57] Roup C.M., Wiley T.L., Wilson R.H. (2006). Dichotic Word Recognition in Young and Older Adults. J. Am. Acad. Audiol..

[58] Ianiszewski A., Fuente A., Gagné J.-P. (2021). Association Between the Right Ear Advantage in Dichotic Listening and Interaural Differences in Sensory Processing at Lower Levels of the Auditory System in Older Adults. Ear Hear..

[59] Weihing J., Musiek F. (2014). The influence of aging on interaural asymmetries in middle latency response amplitude. J. Am. Acad. Audiol..

[60] Ianiszewski A., Fuente A., Gagné J.-P. (2021). Auditory brainstem response asymmetries in older adults: An exploratory study using click and speech stimuli. PLoS ONE.

[61] Behtani L., Fuente A., Ianiszewski A., Al Osman R., Hickson L. (2021). Right-ear advantage for unaided and aided speech perception in noise in older adults. J. Int. Adv. Otol..

[62] Mukari S.Z.-M.S., Wahat N.H.A., Mazlan R. (2014). Effects of ageing and hearing thresholds on speech perception in quiet and in noise perceived in different locations. Korean J. Audiol..

[63] Tadros S.F., Frisina S.T., Mapes F., Kim S., Frisina D.R., Frisina R.D. (2005). Loss of peripheral right-ear advantage in age-related hearing loss. Audiol. Neurootol..

[64] Derleth P., Georganti E., Latzel M., Courtois G., Hofbauer M., Raether J., Kuehnel V. (2021). Binaural Signal Processing in Hearing Aids. Semin. Hear..

[65] Jerger J., Silman S., Silverman C., Emmer M. (2017). Binaural interference: Quo vadis?. J. Am. Acad. Audiol..

[66] McArdle R.A., Killion M., Mennite M.A., Chisolm T.H. (2012). Are two ears not better than one?. J. Am. Acad. Audiol..

[67] Lavie L., Shechter Shvartzman L., Banai K. (2022). Plastic changes in speech perception in older adults with hearing impairment following hearing aid use: A systematic review. Int. J. Audiol..

[68] Ministerio S. (2013). Hipoacusia bilateral en personas de 65 años y más que requieren uso de audífono. Ministerio de Salud.

[69] Fuentes-López E., Galaz-Mella J., Nieman C.L., Luna-Monsalve M., Marcotti A. (2025). Effect of Attitudes Toward Hearing Loss and Hearing Aids on the Risk of Device Abandonment Among Older Adults With Hearing Loss Fitted in the Chilean Public Health Sector. Ear Hear..

[70] Ellinger R.L., Jakien K.M., Gallun F.J. (2017). The role of interaural differences on speech intelligibility in complex multitalker environments. J. Acoust. Soc. Am..

[71] Cameron S., Dillon H., Newall P. (2006). Development and Evaluation of the Listening in Spatialized Noise Test. Ear Hear..

[72] Ghahraman M.A., Ashrafi M., Mohammadkhani G., Jalaie S. (2020). Effects of aging on spatial hearing. Aging Clin. Exp. Res..

[73] Gohari N., Dastgerdi Z.H., Rouhbakhsh N., Afshar S., Mobini R. (2023). Training Programs for Improving Speech Perception in Noise: A Review. J. Audiol. Otol..

[74] Laplante-Lévesque A., Nielsen C., Jensen L.D., Naylor G. (2014). Patterns of hearing aid usage predict hearing aid use amount (data logged and self-reported) and overreport. J. Am. Acad. Audiol..

[75] Solheim J., Hickson L. (2017). Hearing aid use in the elderly as measured by datalogging and self-report. Int. J. Audiol..

[76] Perez E., Edmonds B.A. (2012). A systematic review of studies measuring and reporting hearing aid usage in older adults since 1999: A descriptive summary of measurement tools. PLoS ONE.

