# Supplementary material for: Association Between Unaided Speech Perception in Noise and Hearing Aid Use Mediated by Perceived Benefit
Source: Audiol Res. 2025 May 1;15(3):50. doi: 10.3390/audiolres15030050 (PMC12101150; doi:10.3390/audiolres15030050)
Supplement: Supplementary file 1 [file audiolres-15-00050-s001.zip › audiolres-3570990-supplementary.pdf]

Supplementary Material

Appendix S.1

To conduct a sensitivity analysis of the results, the mediation model was re-estimated using structural equation modeling with partial least squares (PLS-SEM), considering the same variables used in the multivariate ordinal GSEM model (Table S1). Consistent with the main findings, a significant and positive indirect effect was observed in the path from SPiN in the left ear to perceived benefit with HA ($p < 0.001$). Similarly, in alignment with the main results, SPiN in the right ear showed no significant indirect effect on this variable (*p=0.095*). Thus, participants with better SPiN performance in the left ear reported greater perceived benefit with HA, which, in turn, was associated with increased HA use.

Table S1. Estimation of the indirect effect of SPIN on HA use mediated by the perceived benefit of using a HA.[a]

	Indirect Effect[b]	p-value
**Path**		
SPiN OD →Perceived Benefit	-0.220	0.095
SPiN OI →Perceived Benefit	**0.627**	**<0.001**

[a] Standardized path coefficients from partial least squares structural equation models (PLS-SEM).
[b] The PLS-SEM models were controlled for the effects of age (in years), number of cohabitants, months of hearing aid experience, hearing aid implementation laterality and self-efficacy with hearing aids.
